# Cognitive training with adaptive algorithm improves cognitive ability in older people with MCI

**DOI:** 10.1007/s40520-024-02913-5

**Published:** 2025-01-03

**Authors:** Chenxi Li, Meiyun Li, Yunfeng Shang

**Affiliations:** 1 School of Nursing, Yueyang Vocational Technical College, Yueyang, 414000 China; 2 School of Management, Guangzhou Xinhua University, Guangzhou, 510006 China; 3 Rehabilitation Department, Yueyang Central Hospital, Yueyang, 414000 China

**Keywords:** CT, Adaptive algorithm, Cognitive ability, Learning curve, Compensation assumption

## Abstract

Recent discoveries indicating that the brain retains its ability to adapt and change throughout life have sparked interest in cognitive training (CT) as a possible means to postpone the development of dementia. Despite this, most research has focused on confirming the efficacy of training outcomes, with few studies examining the correlation between performance and results across various stages of training. In particular, the relationship between initial performance and the extent of improvement, the rate of learning, and the asymptotic performance level throughout the learning curve remains ambiguous. In this study, older adults underwent ten days of selective attention training using an adaptive algorithm, which enabled a detailed analysis of the learning curve's progression. Cognitive abilities were assessed before and after CT using the Mini-mental State Examination (MMSE) and the Montreal Cognitive Assessment (MoCA). The findings indicated that: (1) Initial performance is positively correlated with Learning amount and asymptotic performance level, and negatively correlated with learning speed; (2) Age is negatively correlated with learning speed, while it is positively correlated with the other three parameters. (3) Higher pre-training MMSE scores predicted higher post-training MMSE scores but less improvement; (4) Higher pre-training MoCA scores predicted higher post-training MoCA scores and less improvement; (5) The parameters of the learning curve did not correlate with performance on the MMSE or MoCA. These results indicate that: (1)Selective attention training using adaptive algorithms is an effective tool for cognitive intervention; (2) Older individuals with poor baseline cognitive abilities require more diversified cognitive training; (3) The study supports the compensation hypothesis.

**Supplementary Information:**

The online version contains supplementary material available at 10.1007/s40520-024-02913-5.

## Introduction

Cognitive training (CT) is a structured program of mental exercises designed to preserve or enhance cognitive abilities that facilitate daily activities and promote independent living [9]. The foundation of CT lies in the brain's neuroplasticity—the ability of its structure and neural pathways to adapt and change [6, 7]. CT has been extensively utilized to mitigate cognitive decline in aging populations [1] and to enhance cognitive function in individuals with mild cognitive impairment (MCI) [11, 21]. Nevertheless, Early intervention during the MCI stage is more effective than improving cognitive status in older individuals with dementia. The MCI stage represents a critical juncture in the gradual progression from normal cognitive function to dementia [8]. Effective intervention during this stage can significantly postpone the advancement of this transition [24].

Older people can benefit significantly from CT, as the brain retains a considerable capacity for plasticity well into old age [20]. An intriguing question arises: who stands to gain the most from CT? This question touches upon the debate between two competing hypotheses—the magnification account [14] and the compensation account [12, 18]. The magnification account proposes that individuals possessing stronger baseline abilities are inclined to reap greater benefits from cognitive training, displaying a trend similar to the Matthew Effect. More precisely, those with higher baseline levels tend to excel in training, showing steeper learning curves and consequently benefiting more from cognitive interventions. Conversely, the compensation account suggests that those with poorer cognitive abilities will benefit more, as their brains have greater potential for learning. Research into this matter could not only deepen our understanding of human plasticity but also illuminate the development of tailored CT protocols.

Upon a careful review of the literature, we discovered that researchers have yet to chart the CT learning curve for the older population diagnosed with MCI. Indeed, the learning curve is crucial for scrutinizing the detailed time course of the learning process, including initial performance, the volume of learning, learning speed, and the asymptotic performance level. By investigating the correlation between these factors, we can elucidate whether individuals with MCI who exhibit better initial performance learn more or less and achieve a superior final performance during CT. We can also determine who learns at a faster or slower pace. Furthermore, this method aids in deepening our understanding of the progression from cognitively unimpaired (CU) to MCI.

In the present study, an adaptive staircase method was employed to monitor the learning process. The threedown/ one-up staircase procedure converged to 79.4% correct responses [10], resulting in a moderate level of difficulty for the cognitive task, neither overly easy nor excessively challenging. According to the universal theory of education, individuals achieve the best learning effects when engaging in tasks at a moderate level of difficulty. From a different perspective, maintaining consistent accuracy in CT can circumvent the ambiguity associated with the trade-off between speed and accuracy

In this study, we examined the impact of CT using an adaptive method on older individuals with MCI. Their cognitive function was evaluated using the Mini-Mental State Examination (MMSE) and the Montreal Cognitive Assessment (MoCA) both before and after CT. Attention serves as a prerequisite for individuals to engage in the majority of tasks, and it can be improved through training [17]. Considering that selective attention occupies a key position within attentional functions, it is selected as the focus of CT.The investigation had two objectives: (1) to differentiate between magnification and compensation theories based on the characteristics of the learning curve, and (2) to observe whether the benefits of selective attention training transfer to other major cognitive functions as assessed by the MMSE and MoCA tests.

## Method

### Subjects

Sixty older individuals diagnosed with MCI (31 males and 29 females, aged between 60 and 95) volunteered to participate in the current study. The inclusion criteria for research subjects with MCI were as follows: (1) Age 60 and above; (2) Normal hearing and vision; (3) MCI patients were diagnosed with MCI in accordance with the standards set by the International MCI Working Group and the diagnostic criteria of the European Alzheimer's Disease Consortium MCI Working Group [16]: a. The primary complaint was memory loss within the past year, while general cognitive function remained normal. b. Did not meet the diagnostic criteria for dementia as outlined in DSM-IV (Diagnostic and Statistical Manual of Mental Disorders, Fourth Edition). c. Based on the Montreal Cognitive Assessment (MoCA), patients with MCI were identified using the evaluation criteria from the study [13]. Specifically, illiterate individuals had a MoCA score of ≤ 14,Those with 1–6 years of education had a MoCA score ≤ 20 points; And those with 7 or more years of education had a MoCA score ≤ 25 points.

Exclusion criteria for research participants: a. Functional or organic mental disorders; b. Language communication barriers; c. Exclusion of other diseases or factors that may lead to a decline in brain function (including physical diseases, depression, stroke, traumatic brain injury, drug and alcohol poisoning, and the use of psychiatric medications); d. Unhealthy behaviors (such as insomnia, alcoholism, etc.). Prior to our study, all participants signed informed consent forms prior to the experiment. The Institutional Review Board of the First People’s Hospital of Yueyang approved the current study, which also adhered to the principles of the Declaration of Helsinki. Sixty participants were randomly assigned to the training (n = 30) and control groups (n = 30). Due to the decision of two participants in the training group to withdraw from the experiment, only twenty-eight remained. The ages of the two groups were comparable (all, P > 0.1).All screenings were conducted collectively by the research team Table 1.

**Table 1:** Basic information for two groups

Item	Training group (*n* = 28)	Control group (*n* = 30)	*χ*^2^	*P* value
Gender Male Female	14 14	16 14	0.064	0.8
Diploma > 7 years < 7 years	9 19	11 19	0.131	0.717
Occupation Mental labor Manual labor	8 20	10 20	0.153	0.695

### Apparatus

The experiment was run by MATLAB with PsychToolbox extensions [2]. The screen has a mean luminance of 41 cd/m2, a refresh rate of 60 Hz, and a resolution of 1366 × 768 pixels.

## Stimuli and procedure

**Fig. 1:**
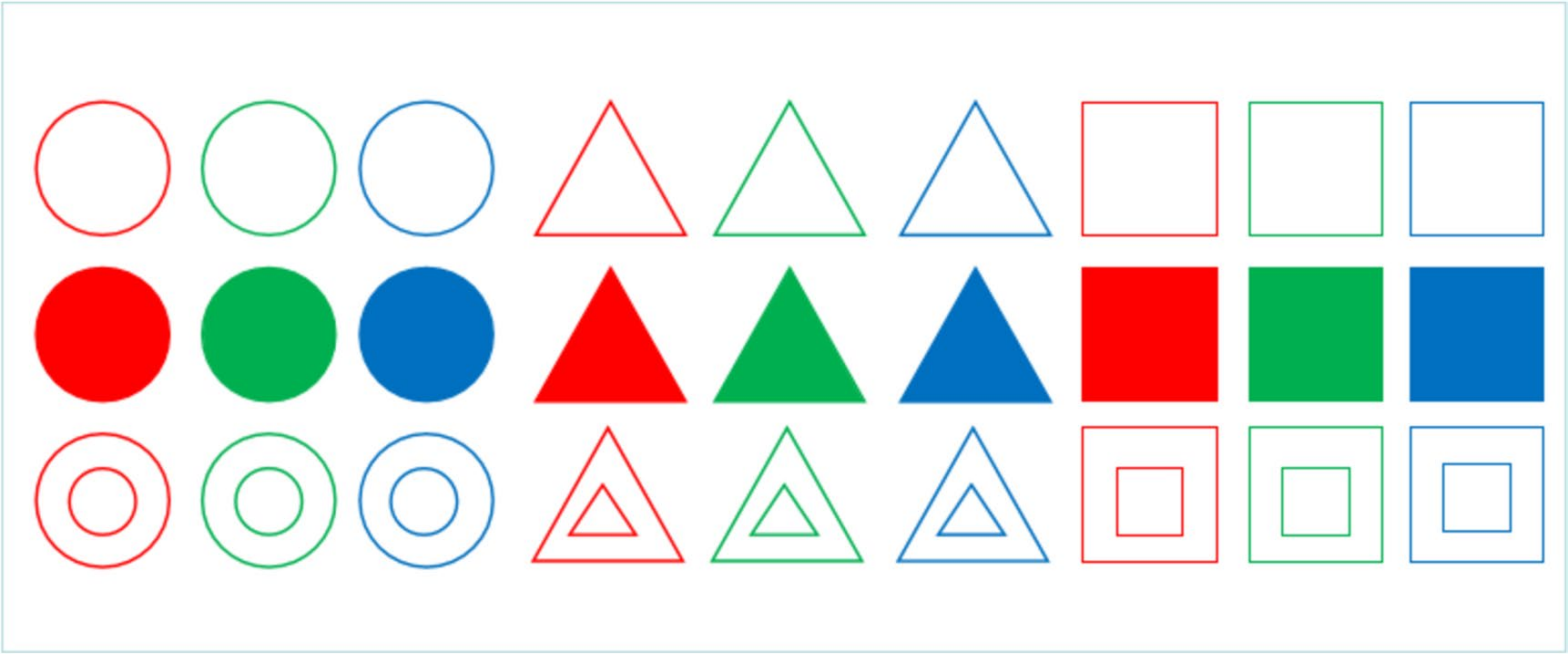
Pictures in selective attention training

The selective attention task comprised twenty-seven images, each featuring one of three colors (red, green, blue), one of three shapes (square, triangle, circle), and one of three interior structures (hollow, nested, solid). Each trial commenced with a 200-ms blank screen, followed by the random selection of two images from the set of twenty-seven to serve as targets. Participants were tasked with determining whether these two images shared the same color (Fig. 1A) or not (Fig. 1B), using the buttons on a game controller. Participants were instructed to disregard the shape and interior structure of the images. A brief beep signaled the accuracy of their response immediately after they responded. Following their response, the targets vanished, and a 500-ms blank screen appeared. Throughout the experiment, a fixation cross remained centered on the screen.

The three down one up staircase method was employed to regulate the display duration of images and monitor the temporal progression of selective attention training, as per the subjects' responses. Within the realm of perceptual learning, the up-down staircase method is commonly utilized to determine the threshold required to attain a particular percentage of correct responses. This is achieved by adjusting the stimulus level (such as contrast, luminance, etc.) according to the subject's responses [23], 25. Prior to training, the experimenter typically establishes an initial stimulus level based on pilot data. Subsequently, the stimulus level is modified, either increased or decreased, using adaptive or fixed step sizes, contingent upon the subject's performance in each trial. The estimated threshold for a series of trials is calculated at intervals of 60 to 80 trials, and the learning curve is charted using these estimated thresholds across several blocks. The method functions by decreasing the presentation time by 10% (to 90% of its previous value) after the subject has made three consecutive correct responses, and by increasing it by 10% (to 110% of its previous value) following each incorrect response. A reversal or end point was defined by the trial after which presentation time was increased or decreased. The initial four (for an even total number of reversals) or five (for an odd total) reversals were omitted. The threshold for distinguishing the color of the two images was determined by averaging the subsequent reversals. The initial presentation duration for each staircase was established close to the anticipated threshold, as inferred from preliminary testing. The mean accuracy rate was 79.3%.

### Design

The experiment was divided into three distinct stages: a pre-test phase, a selective attention training phase, and a post-test phase. During the pre-test, participants' cognitive functions were evaluated using MMSE and MoCA. The training phase consisted of subjects completing 10 days of training over a span of two weeks, with each day's training comprising two separate blocks. Following the training phase, the post-test was conducted, where cognitive functions were once again assessed using the MMSE and MoCA. The training group underwent all three stages, whereas the control group only took part in the pre- and post-tests.

The learning curve was modeled by a single exponential function:

1 $$T(n) = \alpha \times \exp \left( {\frac{ - n}{\beta }} \right) + \gamma$$

where T is the threshold, n is the training blocks. Learning amount, learning speed, and asymptotic performance level was denoted by α, β, and γ, respectively. Thus,α + γ indicated the initial performance. For β, the large value referred to a slow learning speed and vice versa. For α and γ, the large value referred to poor threshold and vice versa.

## Results

### Training group

The exponential function was applied to the block thresholds of each participant. The average goodness of fit (r2) for each participant was 63.52 ± 4.27%, and the mean values for α, β, γ, and α + γ across all participants were 8.29 ± 1.27 (mean ± standard deviation), 8.12 ± 2.01, 1.89 ± 0.11, and 9.48 ± 1.34, respectively. These results suggest that participants in the training group experienced significant learning gains. Subsequently, a Pearson correlation analysis was conducted on the four parameters of the learning curve. Initially, the initial threshold was positively correlated with the amount of learning and the asymptotic performance level, but negatively correlated with learning speed. This suggests that participants who performed poorly baseline Abilities exhibited a greater amount of learning and faster learning speeds, yet had a poorer asymptotic performance level. Secondly, the amount of learning was negatively correlated with learning speed, but positively correlated with the asymptotic performance level. This indicates that a larger amount of learning is associated with faster learning speeds and a poorer asymptotic performance level. Thirdly, learning speed was negatively correlated with the asymptotic performance level, suggesting that participants with faster learning speeds were likely to have a poorer asymptotic performance level. The correlation matrix is detailed in Table 2.

**Table 2:** Correlation matrix

	Age	Moca In pre-test	Moca In post-test	Mmse In pre-test	Mmse In post-test	MoCA gain	MMSE gain	Learning amount	Learning speed	Asymptotic performance level	Initial performance
Age	1	0.311	0.158	0.130	0.191	− 0.327	− 0.029	0.565**	− 0.435*	0.548**	0.584**
Moca in pre-test	0.311	1	0.817**	0.916**	0.756**	− 0.641**	− 0.696**	− 0.069	0.011	− 0.071	− 0.071
Moca in post-test	0.158	0.817**	1	0.783**	0.754**	− 0.080	− 0.506**	− 0.276	0.141	− 0.208	− 0.280
Mmse in pre-test	0.130	0.916**	0.783**	1	0.742**	− 0.541**	− 0.830**	− 0.298	0.153	0.004	− 0.284
Mmse in post-test	0.191	0.756**	0.754**	0.742**	1	− 0.303	− 0.242	− 0.138	0.078	− 0.153	− 0.144
MoCA gain	− 0.327	− 0.641**	− 0.080	− 0.541**	− 0.303	1	0.530**	− 0.248	0.168	− 0.155	− 0.250
MMSE gain	− 0.029	− 0.696**	− 0.506**	− 0.830**	− 0.242	0.530**	1	0.317	− 0.157	− 0.133	0.292
Learning amount	0.565**	− 0.069	− 0.276	− 0.298	− 0.138	− 0.248	0.317	1	− 0.648**	0.541**	0.998**
Learning speed	− 0.435*	0.011	0.141	0.153	0.078	0.168	− 0.157	− 0.648**	1	− 0.466*	− 0.656**
Asymptotic performance level	0.548**	− 0.071	− 0.208	0.004	− 0.153	− 0.155	− 0.133	0.541**	− 0.466*	1	0.596**
Initial performance	0.584**	− 0.071	− 0.280	− 0.284	− 0.144	− 0.250	0.292	0.998**	− 0.656**	0.596**	1

**Fig. 2:**
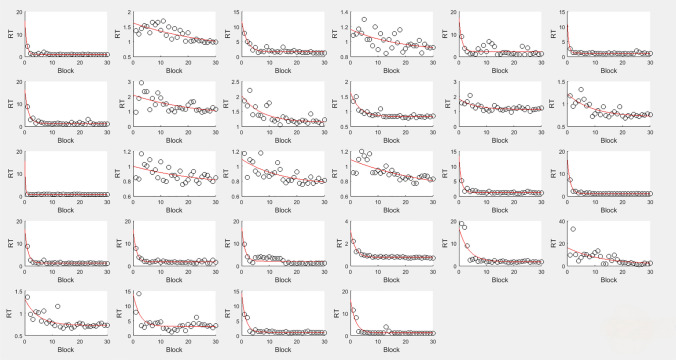
Individual learning curve

Meanwhile, The learning progress of 28 participants is plotted in Fig. 2.

From Tables 3 and 4 the MoCA and MMSE scores were (14.82 ± 4.81) and (20.07 ± 3.67) in the pre-test, respectively; and were (21.36 ± 3.70) and (25.00 ± 2.11) in the post-test, respectively. A paired T-test revealed that the differences between pre- and post-test scores were significant for both MoCA (P < 0.05) and MMSE (P < 0.05). The improvements in MoCA and MMSE scores were (6.39 ± 2.96) and (4.93 ± 2.54), respectively. There was no significant correlation between age and either MoCA or MMSE scores. The MoCA score in the pre-test was positively correlated with that in the post-test, as well as with its improvement. Similarly, the MMSE score in the pre-test was positively correlated with that in the post-test, and with its improvement. Additionally, the improvements in MoCA and MMSE scores were positively correlated. These results suggest that subjects with poor cognitive abilities experienced greater benefits from the selective attention training. Furthermore, the four parameters of the learning curve were not correlated with the scores of MoCA or MMSE in either the pre- or post-tests, nor with their improvements.

**Table 3:** Comparison of MOCA Scores Between Two Groups Before and After the Intervention(mean ± sd)

Group	Participants	Pre-test (MOCA)	Post-test (MOCA)	Average progress	*T* value	*P* value
Experimental	28	14.82 ± 4.81	21.36 ± 3.70	6.39 ± 2.96	12.431	0.000
Control	30	14.00 ± 3.35	15.17 ± 3.24	1.17 ± 1.05	6.067	0.000
*T* value		0.750	6.788	8.833		
*P* value		0.457	0.000	0.000

**Table 4:** Comparison of MMSE scores between two groups before and after the intervention (mean ± sd)

Group	Participants	Pre-test (MOCA)	Post-test (MOCA)	Average progress	*T* value	*P* value
Experimental	28	20.07 ± 3.67	25.00 ± 2.11	4.93 ± 2.54	10.277	0.000
Control	30	19.67 ± 2.73	20.37 ± 2.59	0.70 ± 0.70	5.46	0.000
*T* value		0.474	7.435	8.519		
*P* value		0.638	0.000	0.000		

### Control group

The MoCA and MMSE scores were (14.00 ± 3.35) and (19.67 ± 2.73) in the pre-test, respectively; and were (15.17 ± 3.24) and (20.37 ± 2.59) in the post-test, respectively. A paired T-test revealed that the differences between the pre- and post-test scores were significant for both MoCA (P < 0.05) and MMSE (P < 0.05). However, the improvements in MoCA and MMSE scores were minimal: (1.17 ± 1.05) and (0.70 ± 0.70), respectively.

### Comparison between two groups

The age, MoCA, and MMSE scores in the pre-test were comparable between the two groups (P < 0.05). However, the MoCA scores in the pre-test for the training group were significantly higher than those in the control group (t = 8.833, P < 0.01). The improvement in MMSE scores was also greater for the training group, which were significantly higher than those in the control group (t = 8.519, P < 0.01). These results indicated that the training group gained cognitive ability after the selective attention training, and there was a practice effect on the MoCA and MMSE.

## Discussion

In the present investigation, we assessed the impact of CT utilizing an adaptive approach on selective attention among individuals with MCI. Additionally, we explored its transfer effects on other cognitive functions through the use of MMSE and MoCA assessments.The findings indicated that: (1)Initial performance is positively correlated with Learning amount and asymptotic performance level, and negatively correlated with learning speed; (2) Age is negatively correlated with learning speed, while it is positively correlated with the other three parameters. (3)Higher pre-training MMSE scores predicted higher post-training MMSE scores but less improvement; (4)Higher pre-training MoCA scores predicted higher post-training MoCA scores and less improvement; (5)The parameters of the learning curve did not correlate with performance on the MMSE or MoCA.

Based on the previous discussion, among the four parameters involved in the learning curve, the initial performance is related to baseline ability. A higher initial scores indicates poorer baseline ability of the participants, whereas a lower initial scores suggests better baseline ability. The asymptotic performance level is associated with the final performance that participants can achieve through cognitive learning. A higher asymptotic performance level indicates poorer final performance achieved through cognitive learning, whereas a lower asymptotic performance level suggests better final performance. Learning speed represents the time taken by participants to progress from the initial performance to the final performance through training, while learning amount represents the magnitude of cognitive learning.

Current research shows that the initial performance is positively correlated with learning amount and negatively correlated with learning speed. This indicates that within a certain range, poorer baseline ability leads to a greater amount of learning, and this learning occurs at a faster speed. This suggests that older adults with poorer baseline abilities have a greater need for cognitive training, and the training effects are both rapid and significant. However, the longevity of these benefits is currently uncertain. Nonetheless, this is a path worth pursuing in the future.This outcome strongly supports the compensation hypothesis, which posits that aging individuals with diminished attentional capacities will experience more significant learning gains during training. Our results align with those of previous studies [15, 22]. For instance, Borella et al., [1] discovered that healthy older adults with lower working memory and vocabulary scores prior to testing benefited more from the training program. An advantage of our study is the broad age range of the MCI subjects, spanning from 62 to 95 years. Consequently, our findings are more universally applicable.

The initial performance is positively correlated with the asymptotic performance level. This reflects that older adults with poorer baseline abilities tend to achieve poorer final performances, whereas those with better baseline abilities, despite having smaller learning amounts, can achieve superior final performances through cognitive learning. This implies that older adults with poorer baseline abilities may require more diversified training programs to achieve the same training effects as those with better baseline abilities. Research has also found that age is positively correlated with initial performance, asymptotic performance level, and learning amount, but negatively correlated with learning speed. This suggests that as age increases, cognitive baseline abilities decline. This indicates that age is associated with cognitive levels and, to a certain extent, suggests that the probability of developing Alzheimer's disease increases with age. This result is consistent with some previous neurological studies, such as those by Depp et al., [3], which found age-dependent structural defects in myelin that directly or indirectly promote the formation of Aβ plaques, a risk factor for amyloid deposition.

The Mini-Mental State Examination (MMSE) is a widely used short cognitive screening test, initially developed to detect Mild Cognitive Impairment (MCI) and dementia in medical and psychiatric patients. However, the MMSE and the Montreal Cognitive Assessment (MoCA), a more recent test created in a memory clinic setting, have distinct target functions. The MMSE allocates 24 out of 30 points to memory, language, and orientation, and only 1 point to visuoconstructive function, making it less sensitive to milder forms of cognitive impairment [19]. In contrast, the MoCA dedicates 14 out of 30 points to visuospatial, attentional, and executive functions.

In the current study, both tests were employed to assess the cognitive function of subjects experiencing normal aging. The results indicated that both groups benefited from the tests, suggesting that the transfer effect of selective attention training is robust. A practice effect for the MMSE and MoCA was also noted in the control group of this study, a phenomenon that has been documented in the literature. For instance, [5] observed slight increases in MMSE and MoCA scores after repeated measurements. In the present study, the training group showed improvements of 4.39 and 6.79 on the MMSE and MoCA, respectively, compared to the control group's improvements of 0.70 and 1.17. Consequently, we believe that our observed transfer effect is quite reliable.The reason may be that the brain is an organic whole, and any training of a function is not independent, but requires the close cooperation of several brain regions to complete. This has been verified in neurobiology, such as Engvig et al., [4], who found that cognitive training can change the activation state of some brain regions related to the training task.

In summary, we found that selective attention training with adaptive methods effectively enhanced the selective attention of subjects experiencing MCI, and that this improvement could transfer to other major cognitive abilities measured by the MMSE and MoCA. Furthermore, initial performance was found to predict learning amount, learning speed, and asymptotic performance levels. Subjects with poorer initial performance often learned faster and more extensively, yet their final performance remained lower than those who started with better initial abilities.Looking ahead, we plan to expand our training initiatives to reach a broader audience, including individuals recovering from strokes and those in the early stages of Alzheimer's disease. We will tailor our training programs to various groups, incorporating targeted exercises such as those focusing on executive function and spatial orientation. Once we have established significant outcomes, our aim is to implement more user-friendly approaches, such as creating mobile applications that can be easily accessed on smartphones, thereby facilitating the dissemination of our research findings to communities that lack extensive training resources.

## Supplementary Information

Below is the link to the electronic supplementary material.

Supplementary file1 (DOC 12 KB)

## Data Availability

All data supporting the findings of this study are available within the paper and its Supplementary Information.

## References

[1] Borella E, Carbone E, Pastore M et al (2017) Working memory training for healthy older adults: the role of individual characteristics in explaining short- and long-term gains. Front Human Neurosci. 10.3389/fnhum.2017.00099 10.3389/fnhum.2017.00099 PMC5360719 28381995

[2] Brainard, D. H. (n.d.). The Psychophysics Toolbox Short Title: The Psychophysics Toolbox Corresponding Author. http://color.psych.ucsb.edu/psychtoolbox

[3] Depp C, Sun T, Sasmita AO et al (2023) Myelin dysfunction drives amyloid-β deposition in models of Alzheimer’s disease. Nature 618:349–357. 10.1038/s41586-023-06120-6 37258678 10.1038/s41586-023-06120-6 PMC10247380

[4] Engvig A, Fjell AM, Westlye LT et al (2012) Memory training impacts short-term changes in aging white matter: a longitudinal diffusion tensor imaging study. Hum Brain Mapp 33:2390–2406. 10.1002/hbm.21370 21823209 10.1002/hbm.21370 PMC6870063

[5] Feeney J, Savva GM, O’Regan C et al (2016) Measurement error, reliability, and minimum detectable change in the mini-mental state examination, montreal cognitive assessment, and color trails test among community living middle-aged and older adults. J Alzheimer’s Dis 53:1107–1114. 10.3233/JAD-160248 27258421 10.3233/JAD-160248

[6] Han K, Chapman SB, Krawczyk DC (2020) Cognitive training reorganizes network modularity in traumatic brain injury. Neurorehabil Neural Repair 34:26–38. 10.1177/1545968319868710 31434528 10.1177/1545968319868710

[7] Hosseini SMH, Kramer JH, Kesler SR (2014) Neural correlates of cognitive intervention in persons at risk of developing alzheimer’s disease. Front Aging Neurosci. 10.3389/fnagi.2014.00231 25206335 10.3389/fnagi.2014.00231 PMC4143724

[8] Jack CR, Bennett DA, Blennow K et al (2018) NIA-AA research framework: toward a biological definition of Alzheimer’s disease. Alzheimer’s Demen 14:535–562. 10.1016/j.jalz.2018.02.018 10.1016/j.jalz.2018.02.018 PMC5958625 29653606

[9] Landowska A, Wilson ML, Craven MP et al (2024) Adaptative computerized cognitive training decreases mental workload during working memory precision task: a preliminary fNIRS study. Int J Human Comp Stud. 10.1016/j.ijhcs.2023.103206

[10] Levitt, H. (n.d.). Transformed Up-Down Methods in Psychoacoustics. http://asadl.org/terms 5541744

[11] Li BY, He NY, Qiao Y et al (2019) Computerized cognitive training for Chinese mild cognitive impairment patients: a neuropsychological and fMRI study. NeuroImage Clin. 10.1016/j.nicl.2019.101691 30708349 10.1016/j.nicl.2019.101691 PMC6354286

[12] López-Higes R, Martín-Aragoneses MT, Rubio-Valdehita S et al (2018) Efficacy of cognitive training in older adults with and without subjective cognitive decline is associated with inhibition efficiency and working memory span, not with cognitive reserve. Front Aging Neurosci. 10.3389/fnagi.2018.00023 29456502 10.3389/fnagi.2018.00023 PMC5801297

[13] Lu J, Li D, Li F et al (2011) Montreal cognitive assessment in detecting cognitive impairment in chinese elderly individuals: a population-based study. J Geriatr Psychiatry Neurol 24:184–190. 10.1177/0891988711422528 22228824 10.1177/0891988711422528

[14] Matysiak O, Kroemeke A, Brzezicka A (2019) Working memory capacity as a predictor of cognitive training efficacy in the elderly population. Front Aging Neurosci. 10.3389/fnagi.2019.00126 31214015 10.3389/fnagi.2019.00126 PMC6554703

[15] McDonough IM, Madan CR (2021) Structural complexity is negatively associated with brain activity: a novel multimodal test of compensation theories of aging. Neurobiol Aging 98:185–196. 10.1016/j.neurobiolaging.2020.10.023 33302180 10.1016/j.neurobiolaging.2020.10.023

[16] Mild cognitive impairment as a diagnostic entity. (2004). In J Intern Med (Vol. 256) 10.1111/j.1365-2796.2004.01388.x 15324362

[17] Pozuelos JP, Combita LM, Abundis A (2019) Metacognitive scaffolding boosts cognitive and neural benefits following executive attention training in children. Develop Sci 22:2. 10.1111/desc.12756 10.1111/desc.12756 30257077

[18] Roheger M, Meyer J, Kessler J et al (2020) Predicting short- and long-term cognitive training success in healthy older adults: who benefits? Aging Neuropsychol Cogn 27:351–369. 10.1080/13825585.2019.1617396 10.1080/13825585.2019.1617396 31092117

[19] Siqueira GSA, Hagemann PDMS, Coelho DDS et al (2019) Can MoCA and MMSE Be Interchangeable Cognitive Screening Tools? Gerontol Soc Am, A Systematic Review. 10.1093/geront/gny126 10.1093/geront/gny126 30517634

[20] Toricelli M, Pereira A, Souza Abrao G et al. (2021). Mechanisms of neuroplasticity and brain degeneration: Strategies for protection during the aging process. In Neural Regeneration Research (Vol. 16, Issue 1, pp. 58–67). Wolters Kluwer Medknow Publications. 10.4103/1673-5374.286952 10.4103/1673-5374.286952 PMC7818866 32788448

[21] Wang Z, Zhang H, Wang J et al (2016) P1–258: Tablet-based multi-domain cognitive training increases the parahippocampus volume in patients with amnestic mild cognitive impairment. Alzheimer’s Demen. 10.1016/j.jalz.2016.06.1007

[22] Willis SL, Caskie GIL (2013) Reasoning training in the ACTIVE study: how much is needed and who benefits? J Aging Health 25:43S-64S. 10.1177/0898264313503987 24385639 10.1177/0898264313503987 PMC3882330

[23] Yehezkel O, Sterkin A, Lev M et al (2016) Gains following perceptual learning are closely linked to the initial visual acuity. Sci Rep 6:25188. 10.1038/srep25188 27122254 10.1038/srep25188 PMC4848560

[24] Zhang H, Wang Z, Wang J et al (2019) Computerized multi-domain cognitive training reduces brain atrophy in patients with amnestic mild cognitive impairment. Translat Psych 9:1. 10.1038/s41398-019-0385-x 10.1038/s41398-019-0385-x PMC6355814 30705261

[25] Zhang P, Hou F, Yan FF et al (2018) High reward enhances perceptual learning. J Vis 18:1–21. 10.1167/18.8.11 10.1167/18.8.11 PMC6108453 30372760

