# Supplementary material for: Cognitive training with adaptive algorithm improves cognitive ability in older people with MCI
Source: Aging Clin Exp Res. 2025 Jan 3;37(1):20. doi: 10.1007/s40520-024-02913-5 (PMC11698878; doi:10.1007/s40520-024-02913-5)
Supplement: Supplementary file 1 — Supplementary file1 (DOC 12 KB) [file 40520_2024_2913_MOESM1_ESM.doc]

**Supplementary Information**

1.This research was supported by Natural Science Foundation of Hunan Province (2022JJ70043 to YS), From Yunfeng Shang，Supported by the Science and Technology Department of Hunan Province, China

2.This research was supported by Excellent youth funding of Hunan Provincial Education Department (22B1076 to CL), From Chenxi Li，Supported by the Education Department of Hunan Province, China
